# Supplementary material for: Protein Condensate Atlas from predictive models of heteromolecular condensate composition
Source: Nat Commun. 2024 Jul 10;15:5418. doi: 10.1038/s41467-024-48496-7 (PMC11237133; doi:10.1038/s41467-024-48496-7)
Supplement: Supplementary file 1 — Supplementary Information [file 41467_2024_48496_MOESM1_ESM.pdf]

Protein Condensate Atlas from predictive models of heteromolecular condensate composition – Supporting Figures

Kadi L. Saar,[1,2,*] Rob M. Scrutton,[2,3] Kotryna Bloznelyte,[1] Alexey S. Morgunov,[2] Lydia L. Good,[2,4] Alpha A. Lee,[5] Sarah A. Teichmann,[5,6] Tuomas P.J. Knowles,[2,5,*]

[1] *Transition Bio Ltd, Cambridge, UK*

[2] *Yusuf Hamied Department of Chemistry, University of Cambridge, Cambridge CB2 1EW, UK*

[3] *Department of Chemistry, University of Oxford, Oxford OX1 3TA, UK*

[4] *Laboratory of Chemical Physics, National Institute of Diabetes and Digestive and Kidney Diseases, National Institutes of Health, Bethesda, MD 20892, USA*

[5] *Cavendish Laboratory, Department of Physics, University of Cambridge, Cambridge CB3 0HE, UK*

[6] *Wellcome Sanger Institute, Wellcome Genome Campus, Hinxton, Cambridge, UK*

[*] To whom correspondence should be addressed: Tuomas P.J. Knowles (tpjk2@cam.ac.uk) and Kadi L. Saar (ksaar@transitionbio.com)

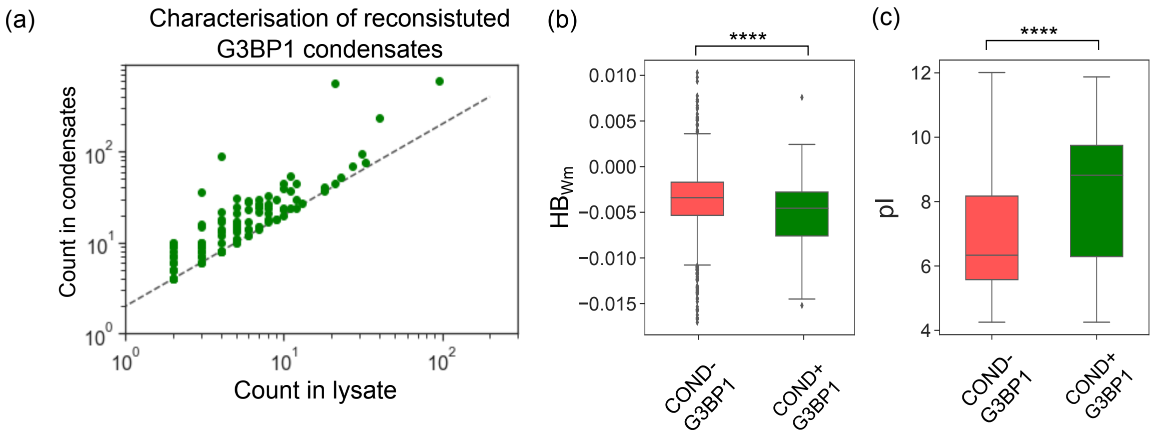

Supplementary Figure S1. Analysis of the composition of reconstituted G3BP1 condensates as characterised by Freibaum et al. 2021 (Main Text). **(a)** The abundance of proteins in the reconstituted G3BP1 condensate correlates with their abundance in the condensate fraction (R^2 = 0.77; cf. R^2 = 0.60 for the NPM1 reconstituted condensates (Main Text)). **(b)** Molecular weight normalised hydrophobicity (Materials and Methods) and **(c)** pI differ significantly ($p < 0.001$) between the proteins that are enriched into the condensate fraction (COND+ G3BP1; 289 proteins) and these that are not enriched (COND- G3BP1; 2669 proteins). As for the NPM1-condensates, only proteins with expression value above 3.5 $\log_{10}$ units were considered for the analysis.

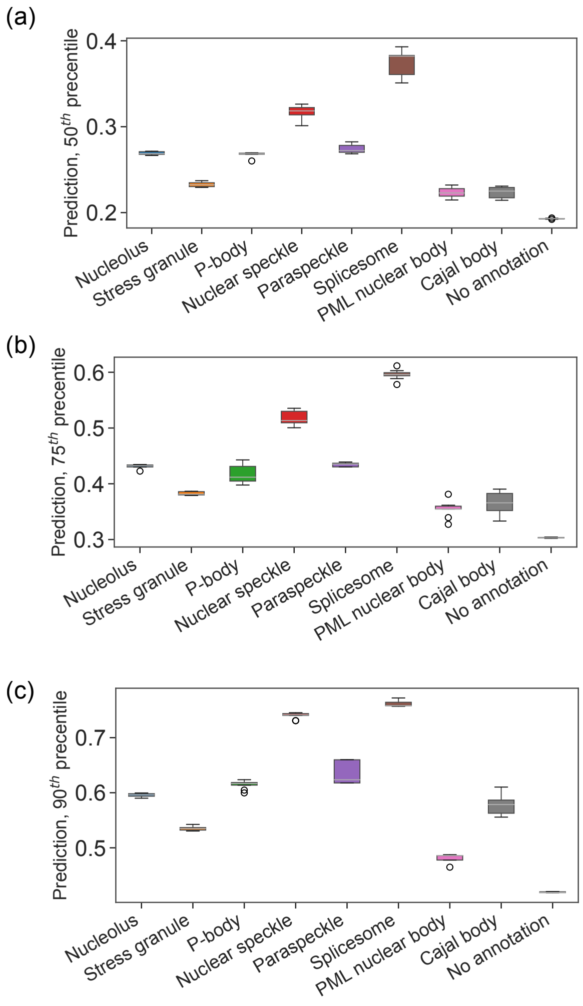

Supplementary Figure S2. **(a)** Median (50th percentile), **(b)** 75th percentile and **(c)** 90th percentile score for proteins in different MLOs as described in the PhaSepDB database and for proteins with no MLO annotation (right-most column). Error bars for each of the percentiles are calculated by randomly sampling 90% of the datapoints ten times and estimating the standard deviation of the distribution. Full distributions of the scores for the MLOs are shown in Figure 5a. The data highlight that the predicted scores for the proteins within any one of these condensate systems are higher than the scores for proteins with no MLO annotation.

(a)

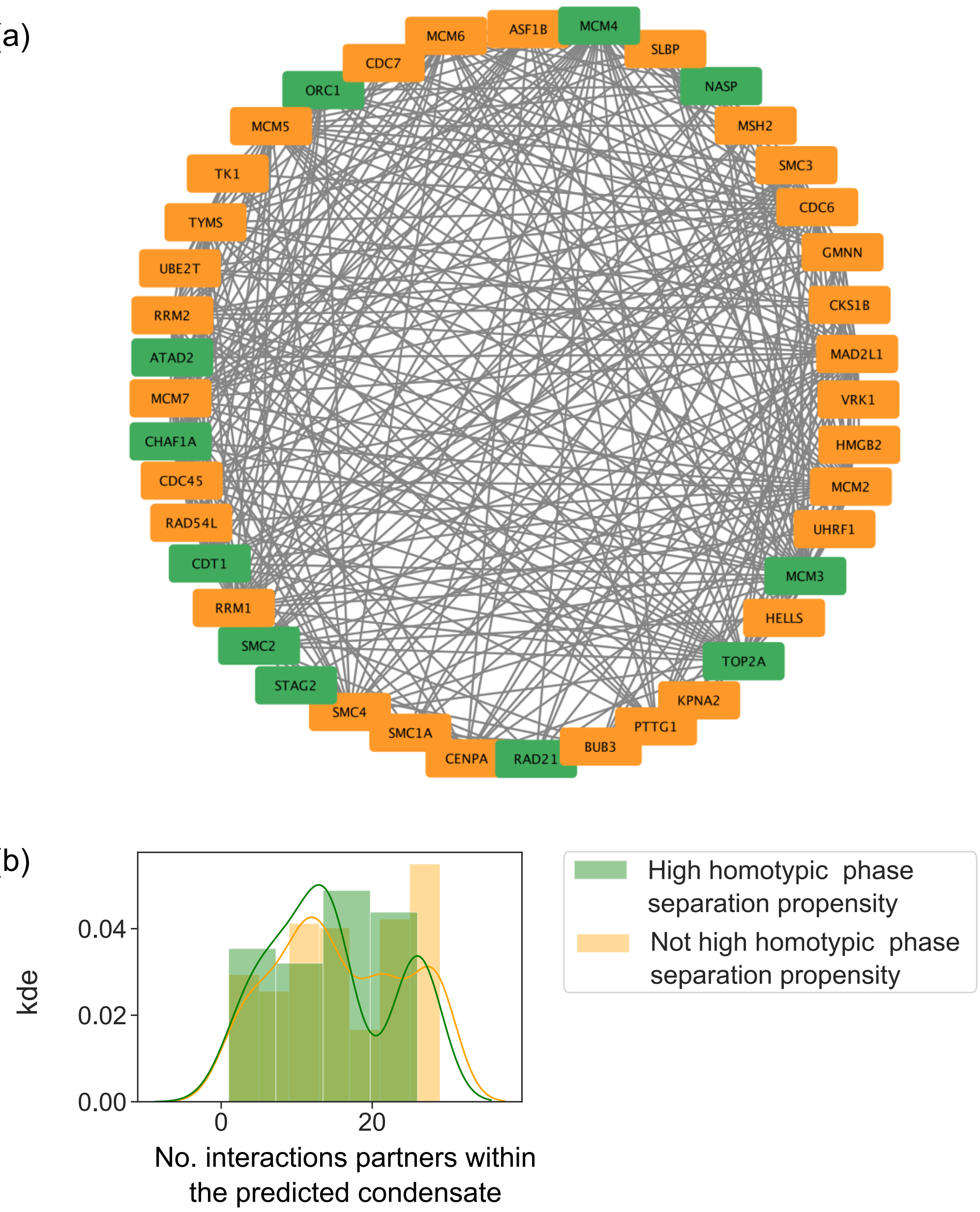

(b)

Supplementary Figure S3. **(a)** Visualisation of reported confident protein-protein interactions (StringDB data; Materials and Methods) within an exemplary condensate cluster in the Protein Condensate Atlas (cluster no. 66, Supplementary Dataset 8). Proteins labelled green have a high predicted homotypic phase separation propensity, proteins labelled orange do not have a high homotypic phase separation propensity but are predicted to localise into condensates. **(b)** Both types of proteins have a comparable number of interaction partners within the predicted condensate cluster. Globally, the proteins that do not have a high homotypic phase separation propensity but localise into condensate regardless have a slightly higher number of interaction partners within the condensate cluster (Figure 5d).

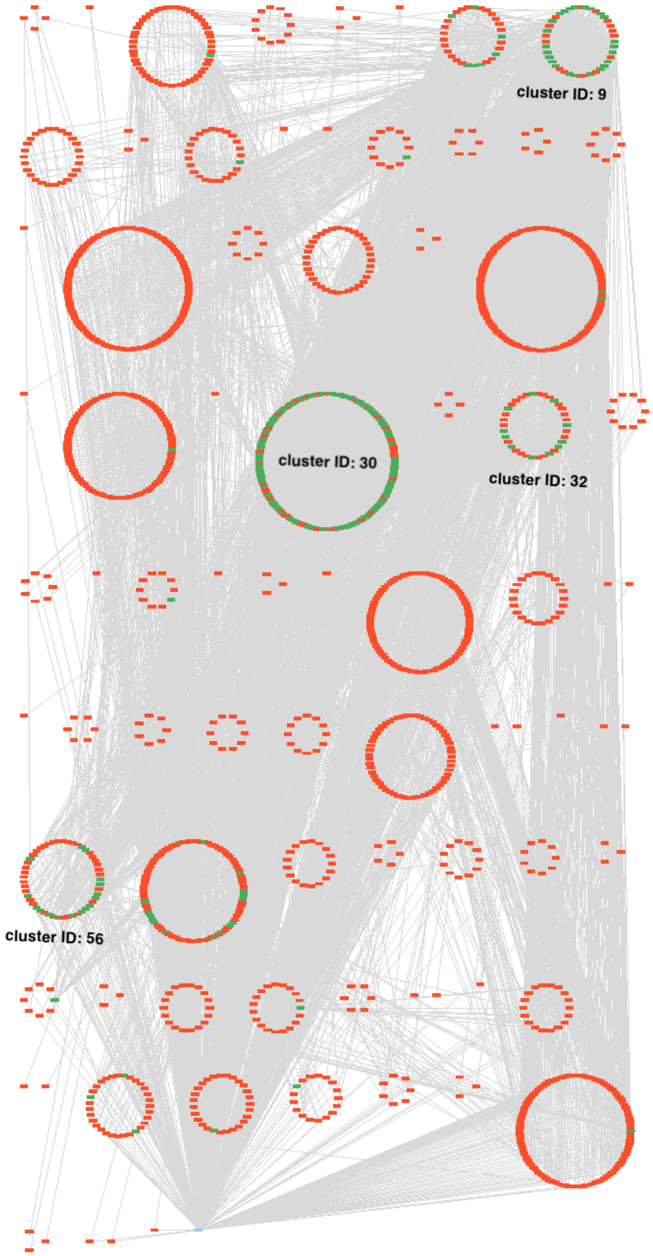

Supplementary Figure S4. Distribution of the proteins that have been reported to localise into spliceosomes across the predicted Protein Condensate Atlas. The proteins that have experimentally been observed in spliceosomes are coloured green. The red proteins within the highlighted clusters with high enrichment scores are candidates for additional proteins that may localise into spliceosomes. Full data is accessible in Supplementary Dataset 8. The visuals are in agreement with high enrichment values highlighted in Figure 6b for splicesome.

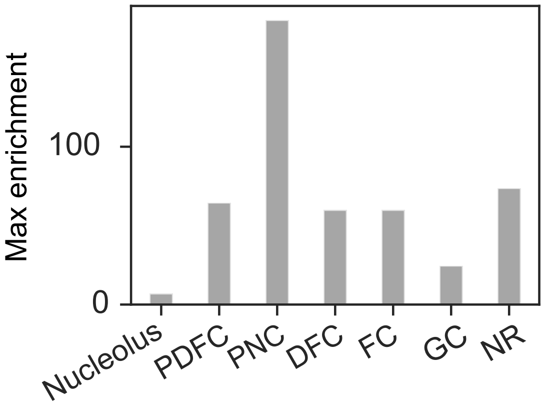

Supplementary Figure S5. The maximum enrichment factor for the nucleolus and the regions identified within it (Shan et al. 2023, Main Text; NR – nuclear rim, FC – fibrillar center, DFC – dense fibrillar component, GC – granular component, PNC – peri-nucleolar compartment; PDFC – periphery of DFC).
