# Supplementary material for: Protein Condensate Atlas from predictive models of heteromolecular condensate composition
Source: Nat Commun. 2024 Jul 10;15:5418. doi: 10.1038/s41467-024-48496-7 (PMC11237133; doi:10.1038/s41467-024-48496-7)
Supplement: Supplementary file 3 — Description of Additional Supplementary Files [file 41467_2024_48496_MOESM3_ESM.pdf]

Supplementary Dataset 1. Characterisation of the lysate and the condensate fraction of reconstituted NPM1 condensates with mass spectrometry for all genes with enrichment values above 2 as obtained by Freibaum et al. 2021 The enrichment value is the ratio between the spectra counts in the two fractions.

Supplementary Dataset 2. Number of copies of each protein (referred to via Uniprot ID) in the U2OS cell line as obtained by Beck et al. 2021.

Supplementary Dataset 3. Proteins (Uniprot IDs) that have been experimentally shown to undergo phase separation on their own as characterised by the PhaSepDB database.

Supplementary Dataset 4. Sequences of folded proteins extracted from the PDB and filtered for sequence diversity as described in Saar et al. 2021, PNAS.

Supplementary Dataset 5. Most enriched GO-terms in the clusters where RNA-driven interactions play a key role (Figure 3d, orange).

Supplementary Dataset 6. Most enriched GO-terms in the clusters where protein-specific interactions play a key role (Figure 3d, pink).

Supplementary Dataset 7. Top 10 highest and lowest predicted proteins by the condensate localisation model and their experimental condensate localisation based on the data in the Human Protein Atlas.

Supplementary Dataset 8. fold. Clusters in the Atlas that are enriched for a certain MLO may include additional proteins that can be find within these MLOs.

Supplementary Dataset 9. The core of the stree granule condensate as characterised by Jain et al.

Supplementary Dataset 10. Calculated enrichment values for all the condensate clusters in the predicted Atlas with respect to known MLOs characterised in the PhaSepDB and regions of the nucleolus.

Supplementary Dataset 11. The composition of regions within the nucleolus as characterised by Shin et al. 2023.
